# Peer bonds and nature’s embrace: exploring the influence of pet caregiving on social well-being and nature connection among Taiwanese children

**DOI:** 10.3389/fpubh.2024.1431939

**Published:** 2024-10-21

**Authors:** Tzuhui Angie Tseng, Hsiao-Yen Fang, Ching-Cheng Shen, Yun-Chen Chang

**Affiliations:** ^1^Department of Environmental and Cultural Resources, National Tsing Hua University, Hsinchu, Taiwan; ^2^Yuansheng Elementary School, Taoyuan, Taiwan; ^3^Graduate Institute of Tourism Management, National Kaohsiung University of Hospitality and Tourism (NKUHT), Kaohsiung, Taiwan; ^4^School of Nursing and Graduate Institute of Nursing, China Medical University, Taichung, Taiwan; ^5^Nursing Department, China Medical University Hospital, Taichung, Taiwan

**Keywords:** children, pets, natural bond, interpersonal relationships, sense of well-being

## Abstract

This study investigated the associations between pet-ownership on nature engagement, interpersonal relationships, and well-being among 471 Taiwanese children aged 11–12, across different genders. The findings revealed that interactions with pets, primarily in the form of caregiving, enhanced empathy toward nature, notably among female participants from various racial and ethnic backgrounds, although direct contact with nature was generally limited. In terms of interpersonal relationships, family bonds were significant, with boys reporting stronger connections. Children who had previously or were currently owning pets displayed increased empathy toward the natural environment. The study highlighted a significant predictive relationship between children’s connection to nature and their development of interpersonal relationships, with peer relationships being particularly influential in predicting children’s well-being. These results underscored the importance of pet caregiving and peer interactions in the emotional and social development of children.

## 1 Introduction

With changes in household structures and demographic patterns, the practice of keeping pets within families has become increasingly widespread. Elevating animals to the category of “pets” involves three distinct characteristics: allowing them indoors, giving them names, and refraining from consuming them (1). According to statistics from 2023, the registered population of dogs and cats in Taiwan has reached 2.22 million, with an estimated 100,000 pet dogs (2). Among these, dogs and cats dominate, while the trend of pet fish ownership is gradually on the rise, emerging as a new choice for many individuals seeking companion animals.

In recent years, the relationship between humans and nature has gained significant attention. Nature-deficit disorder (NDD) refers to a lack of connection with nature that can even alter urban individuals’ behavior and thought patterns (3, 4). Animals are an integral part of the natural world, and interacting with animals is considered one way to reestablish a connection with nature (5). Caring for animals has been shown to divert the attention of hospitalized children outward, reducing anxiety and enhancing parental satisfaction, further facilitating a connection with the world (6). Pet therapy has also been found to reduce pain and control blood pressure in hospitalized children and adolescents (7). Therefore, whether pet ownership can serve as a bridge between humans and nature is one of the motivations behind this study.

Since the Neolithic period, humans have maintained a close relationship with animals (8). In modern times, many individuals have experience in pet ownership, and compared to interactions with wild animals or school pets, interactions and bonding between household pets and children are more frequent. Children who enjoy interacting with animals seem to exhibit better performance in inter-personal relationships and show more interest in campus fauna and flora [as translated by Fan et al. (9)]. Melson (10) also proposed that animals have a significant impact on children’s social development. Animals naturally attract and arouse curiosity in many children, leading them to explore, observe, touch, or converse with animals. Across various ages, children can establish strong emotional connections with animals, easily sharing their fears, joys, setbacks, and daily life experiences with them (11–13). However, previous research has rarely explored whether there is an association between the degree of pet ownership and interaction among children and their interpersonal relationships. This serves as the second motivation for this study.

Our study focuses on children (14) in the 5th and 6th grades, a choice informed by Piaget’s stages of cognitive development (1). According to Piaget, children at this age typically transition from the Concrete Operational Stage to the Formal Operational Stage, marking a critical phase in their cognitive and emotional development. Concrete Operational Stage (7 to 11 years): At this stage, children develop logical thinking skills but are generally focused on concrete situations. Their ability to understand and empathize with others improves, making them more adept at forming relationships (2). These abilities are crucial for our study as they enable children to interact meaningfully with pets, potentially affecting their emotional and social development. Formal Operational Stage (11 years and beyond): As children enter this stage, they begin to think abstractly and are capable of hypothetical and deductive reasoning (2). This allows them to conceptualize and appreciate deeper emotional connections and understand complex aspects of relationships, including those with pets. At this stage, children can reflect on their relationships with pets more profoundly, considering how these relationships influence their emotional state and interpersonal skills.

In addition to being intimate companions of humans, pets can reduce the occurrence of negative emotional expression in children and contribute to the development of expressive abilities (15, 16). Many children include their household pets when drawing pictures of their families, often placing the pets in prominent positions at the forefront or center. Survey reports from the United States also indicate that 60% of dog owners and 54% of cat owners buy gifts for their beloved pets on occasions such as their birthdays or other celebrations [as translated by Fan et al. (9)]. Research suggests that family values often influence children’s sense of well-being through intangible social resources such as interpersonal relationships, parenting quality, mental health, and companionship (17). In recent years, pets have increasingly been considered as family members, playing significant roles within the household (18, 19). This evolving perception is highlighted by innovative efforts such as the development of the cAMpanion ambient display in Taiwan, which aims to enhance the connection between distant pet owners and their dogs by showing the pet’s status through different light colors in real-time, thereby significantly reducing owners’ negative emotions and increasing their perceived closeness (20). This study aims to explore whether interactions between children and pets could also influence family and individual well-being, serving as the third motivation for this research. The quantitative hypotheses of this study are as follows:

*H1*: There are significant differences in nature connection among children based on their pet ownership status (never owned, previously owned, currently own).

*H2*: There are significant differences in interpersonal relationships among children with different pet ownership statuses.

*H3*: There are significant differences in children’s sense of well-being based on their different pet ownership experiences.

*H4*: There is a positive association between children’s connection to nature and their interpersonal relationships.

*H5*: Children’s connection to nature positively affects their sense of well-being.

To complement these hypotheses, qualitative research questions focus on exploring the subjective experiences of children in their interactions with pets, examining how these relationships shape their emotional and social development. By integrating qualitative insights with quantitative findings, the study aims to provide a richer, more nuanced understanding of the dynamic interactions at play.

## 2 Materials and methods

### 2.1 Research design

**Figure 1:**
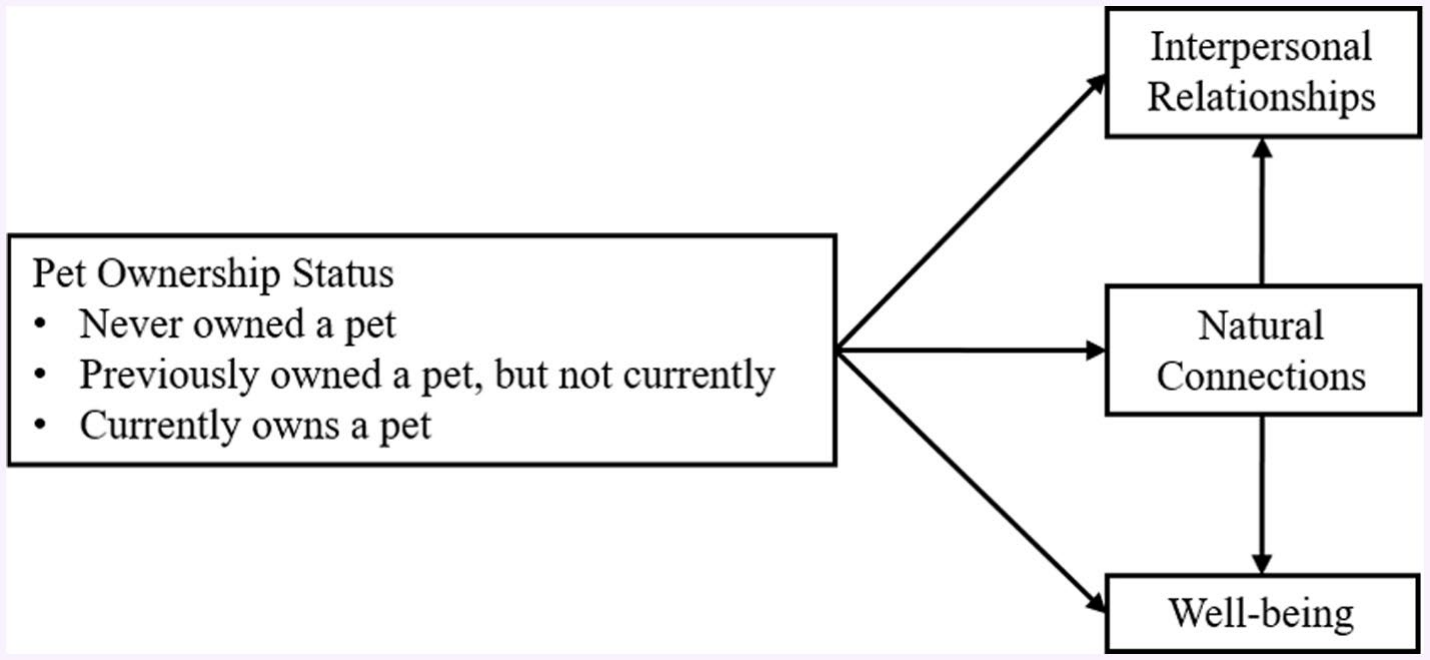
Research framework.

This study adopted a cross-sectional research design and was carried out in Northern Taiwan. We utilized a cross-sectional methodology to gather quantitative data via a survey questionnaire focusing on children and their interactions with pets. Based on the research objectives and hypotheses, the framework of this study is illustrated in Figure 1.

Surveys were distributed and completed in person at schools to facilitate assistance and ensure a higher completion rate, with participants completing them during school hours under teacher supervision. Informed consent was obtained from parents or guardians, and assent was secured from children, ensuring they understood their voluntary participation. The study employed a mixed-methods approach, with participants first completing quantitative questions followed by qualitative ones in the same sitting, typically lasting 30 to 45 min. No follow-ups were conducted, and while participants were not compensated, all schools received a small grant for resources as appreciation for their collaboration.

### 2.2 Participants and sampling

This study targeted fifth and sixth graders from various elementary schools across Taoyuan County, Taiwan. Participants were selected based on their capability to independently care for pets and complete written surveys. To achieve a representative sample, the schools were categorized into four distinct groups based on key demographic factors including socioeconomic status, urban versus rural location, and school size. This categorization was intended to ensure that the sample represented a wide range of environmental contexts, which might influence the relationship between children and their pets.

Definition of the Four Distinct Groups: The stratification within each group was based on the aforementioned demographic factors. Each category was designed to represent different strata within the population of Taoyuan County, enabling us to examine potential variances in pet interaction across diverse backgrounds.

Number of Schools Sampled: A total of 20 schools were sampled from across Taoyuan County. These schools were selected to equally represent the four groups, with five schools from each category.

Opportunity for Student Participation: Not all students at each school were given the opportunity to participate. Instead, within each school, we employed a random sampling technique to select two classes from each of the 5th and 6th grades. This approach ensured that our sampling method was both manageable and within the ethical guidelines set by our institutional review board.

Inclusion and Exclusion Criteria: The inclusion criteria required participants to be either in the 5th or 6th grade and have some form of regular interaction with a pet, either through ownership or frequent contact. Exclusion criteria included students who had reported allergies to animals or those who, due to other reasons specified by their guardians, were not permitted to interact with pets.

The sample size was determined using Huang's (21) formula, targeting a sampling error of less than 5% (e < 0.05) with a 95% confidence level. Based on this calculation, a minimum of 385 participants was required. To account for potential non-responses and ensure adequate representation, 487 questionnaires were distributed. Ultimately, 471 valid questionnaires were collected, providing a sample that accurately represents the diverse student population across the predetermined strata.

### 2.3 Main research tools

#### 2.3.1 Participants characteristics

This section included information about the children’s school, gender, grade, living environment, frequency of park visits, and pet ownership at home. It encompassed whether they owned pets, details about the pets (type, source, role), and parental education levels.

#### 2.3.2 Child-nature connection

The Child-Nature Connection Index Scale, developed by Monroe and Cheng (22), was utilized to assess the connection between children and nature. This scale demonstrated good internal consistency, with a Cronbach’s *α* of at least 0.710 (3). It is divided into four sections: (1) Enjoyment of Nature (7 items), (2) Emotional Affinity to Living Things (4 items), (3) Sense of Oneness with Nature (3 items), and (4) Sense of Responsibility (3 items). Due to similarities between items 7 and 14, which fall under “Enjoyment of Nature” and “Sense of Oneness with Nature” respectively, item 14 was removed, resulting in a total of 16 items. Responses are recorded on a five-point Likert scale ranging from 1 to 5, where higher scores indicate a stronger connection to nature.

#### 2.3.3 Child interpersonal relationships

The Child Interpersonal Relationship Scale developed by Lo (23) was used to assess child interpersonal relationships. The scale covered three major dimensions: “Family Relationships, ““Teacher-Student Relationships, “and “Peer Relationships.” The Cronbach’s *α* coefficient for the overall scale internal consistency was 0.885, and each dimension exhibited a coefficient of 0.8 or higher, indicating good internal consistency and reliability. The scale consisted of 19 items, with responses scored on a five-point Likert scale (1 to 5), where higher scores indicated better interpersonal relation-ships.

#### 2.3.4 Child well-being

The “Stirling Children’s Well-being Scale” (SCWBS), developed by the Stirling Council Educational Psychology Service in 2015, was employed to measure child well-being and demonstrated high internal reliability (*α* = 0.82), good external reliability (α = 0.75), and strong construct validity (*r* = 0.75; 4). Originally comprising 15 items, items 2, 7, and 13 were removed as they did not contribute effectively to the measurement of well-being, reducing the scale to 12 items. Responses are gathered using a five-point Likert scale ranging from 1 to 5, where higher scores indicate greater well-being.

#### 2.3.5 Permission to reuse and copyright

Permission must be obtained for use of copyrighted material from other sources (including the web). Please note that it is compulsory to follow figure instructions.

### 2.4 Interview content design

The qualitative questions were carefully developed based on the theoretical framework established by previous research highlighting the significance of pet-human interactions in child development (24). These questions aimed to explore deeper insights into the children’s personal experiences and feelings toward their pets, which quantitative data alone could not fully capture.

For the qualitative part of our study, a subset of 20 participants was selected from the larger survey group. These participants were chosen based on their responses indicating particularly high or low levels of interaction with pets. The interviews lasted on average 30 min, providing a detailed view into the participants’ emotional and social experiences with their pets.

The data collection was carried out by two trained interviewers who had previous experience in qualitative research methods. They received specific training for this study which included understanding the scope of the study, ethical considerations, and techniques for encouraging children to express themselves more freely and clearly during the interviews.

Prior to commencing the interviews, informed consent was obtained from all participants. The interviews were audio-recorded for accuracy, subsequently transcribed, and analyzed in depth. A semi-structured format was used, allowing participants not only to respond to specific questions but also to elaborate on particular topics, thereby enriching the understanding of their views and experiences. The interview questions are detailed in the Supplementary Table S1. All analyses post-interview was conducted anonymously to ensure confidentiality.

### 2.5 Statistical analysis

Our study employed SPSS 23.0 to conduct descriptive statistics, factor analysis, one-way ANOVA for Hypotheses 1–3 to explore differences in natural connectedness and interpersonal relationships, and regression analyses for Hypotheses 4–5 to examine the impact of pet interaction behaviors, incorporating age, gender, pet type, and interaction frequency as control variables. Missing data were addressed through multiple imputation to avoid biases associated with listwise deletion. For the qualitative component, initial codes were generated and refined through iterative review by two independent coders, with discrepancies resolved through discussion, utilizing qualitative analysis software to manage and categorize the data, ensuring the methodology’s transparency and replicability.

## 3 Results

### 3.1 Sample descriptive statistics

The sample size was 471 participants, of which 22 (4.7%) attended schools with 12 or fewer classes; 61 (12.9%) were in schools with 13–24 classes; 202 (42.9%) in schools with 25–54 classes; and 186 (39.5%) in schools with more than 55 classes. The majority of the participants were from medium to large schools. The number of fifth graders was 384 (81.5%), and sixth graders were 87 (18.5%). Among them, there were 245 boys (52.0%) and 226 girls (48.0%). Regarding the family living environment, 62 (13.1%) lived in apartments, 23 (4.9%) in townhouses, 183 (38.9%) in community buildings, and 203 (43.1%) in detached houses. The majority resided in community buildings and detached houses. Of the students surveyed, 144 (30.6%) had never owned a pet; 136 (28.9%) had previously owned a pet but not currently; and 191 (40.5%) currently had pets at home. Among the respondents, a total of 327 had experience with pet ownership, either in the past or presently. Of these, 132 (28.0%) had one pet, and 56 (11.9%) had two pets.

### 3.2 The association between children’s pet ownership experience on nature connection, interpersonal relationships, and well-being

#### 3.2.1 Analysis of the differences in children’s natural connection with pets

Table 1 illustrated the differences in nature connection based on children’s pet ownership experiences. The results indicated a significant variation in the ‘Empathy for Nature’ aspect of nature connection among children who currently own pets, those who previously owned pets, and those who have never owned pets. Specifically, children who currently own pets and those who previously owned pets have higher scores in the ‘Empathy for Nature’ dimension compared to children who have never owned pets.

**Table 1:** One-way ANOVA of children’s pet-nature connection.

Variable facet	Variable facet	Content	N	M	SD	df	*F*-value	*p*-value
Natural connection	Contact with the natural environment	Never had a pet	144	3.11	0.93	2	0.35	0.71
		Once raised, but not currently	136	3.15	0.98	2		
		Currently being raised	191	3.20	1.06	2		
	Empathize with the natural environment	Never had a pet	144	3.59	0.97	2	13.63***	<0.001
		Once raised, but not currently	136	3.96	0.80	2		
		Currently being raised	191	4.11	0.97	2		
	Cognition of natural environment	Never had a pet	144	3.44	0.93	2	0.81	0.45
		Once raised, but not currently	136	3.63	1.78	2		
		Currently being raised	191	3.54	1.00	2		

***p < 0.001.

Tukey HSD *post hoc* test comparisons further indicated significant differences between specific groups regarding ‘Empathy for Nature.’ Children who previously owned pets but no longer do scored significantly higher in ‘Empathy for Nature’ compared to children who have never owned pets (mean = 3.96, *p* < 0.05). Likewise, children who currently own pets scored significantly higher in ‘Empathy for Nature’ than those who have never owned pets (mean = 4.11, *p* < 0.05). These results suggest that pet ownership, whether past or present, is associated with a heightened intrinsic love for nature. This intrinsic connection appears as an internalized affection toward nature, rather than being manifested through explicit external behaviors.

#### 3.2.2 Analysis of differences in interpersonal relationships among children who keep pets

As indicated in Table 2, there were no significant differences in the three aspects of interpersonal relationships due to children’s pet-keeping behavior.

**Table 2:** Interpersonal relationships among children with pet-keeping experiences.

Variable facet	Variable facet	Content	N	M	SD	df	*F*-value	*p*-value
Interpersonal relationship	Teacher-student relationship	Never owned a pet	144	3.56	1.03	2	1.06	0.35
		Previously owned, currently without	136	3.38	0.98	2		
		Currently owning a pet	191	3.49	1.04	2		
	Peer relationships	Never owned a pet	144	4.08	0.95	2	0.32	0.73
		Previously owned, currently without	136	4.02	0.91	2		
		Currently owning a pet	191	3.99	1.03	2		
	Family relationships	Never owned a pet	144	4.18	1.05	2	0.12	0.88
		Previously owned, currently without	136	4.14	1.36	2		
		Currently owning a pet	191	4.21	1.44	2		

#### 3.2.3 Analyzing the variations in well-being among children with pet-keeping experiences

Furthermore, as shown in Table 3, children’s pet-keeping behavior did not significantly affect the well-being aspect.

**Table 3:** Children’s well-being related to pet-keeping experiences.

Variable facet	Variable facet	N	M	SD	df	*F*-value	*p*-value
Children’s well-being	Never owned a pet	144	3.58	0.81	2	0.70	0.50
	Previously owned, currently without	136	3.50	0.79	2		
	Currently owning a pet	191	3.61	0.98	2		

### 3.3 The association between nature connection, interpersonal relationships, and well-being

#### 3.3.1 The influence of nature connection on children’s interpersonal relationships

Table 4 shows that nature connection significantly affects children’s interpersonal relationships in three areas: teacher-student, peer, and family (*F* values = 54.553, 29.657, and 10.737 respectively, all *p* < 0.001). This connection explains 25.6, 15.7, and 6% of the variance in these areas. Key findings include that both ‘contact with’ and ‘empathy toward’ natural environments positively correlate with better relationships in all three areas, with significant regression coefficients (*β* = 0.319 and *β* = 0.204 for teacher-student; *β* = 0.238 and *β* = 0.214 for peer; *β* = 0.137 and *β* = 0.082 for family, all *p* < 0.05). ‘Awareness of natural environments, ‘however, did not show a significant correlation in any area (*p* > 0.05). The results underscore the importance of nature connection in enhancing various aspects of children’s interpersonal relationships.

**Table 4:** Nature connection on children’s interpersonal relationships.

Variable facet	Independent variable	Unstandardized regression coefficient (B)	S.E	Regression coefficient (β)	*t*	*p*-value	VIF
Teacher-student relationship	Constant	1.375	0.186		7.412***	<0.001	
	Nature connection						
	Contact with natural environments	0.325	0.055	0.319	5.941***	<0.001	1.81
	Empathy toward natural environments	0.219	0.057	0.204	3.860***	<0.001	1.75
	Awareness of natural environments	0.064	0.035	0.079	1.849	0.07	1.15
	*F*-value = 54.553***	*R* = 0.511	R^2^ = 0.261	Adj. R = 0.256			
Peer relationships	Constant	2.517	0.188		13.389***	<0.001	
	Nature connection						
	Contact with natural environments	0.230	0.056	0.238	4.145***	<0.001	1.80
	Empathy toward natural environments	0.217	0.057	0.214	3.792***	<0.001	1.74
	Awareness of natural environments	−0.016	0.035	−0.021	−0.456	0.65	1.14
	*F*-value = 29.657***	*R* = 0.403	R^2^ = 0.162	Adj. R = 0.157			
Family relationships	Constant	2.893	0.269		10.774***	<0.001	
	Nature connection						
	Contact with natural environments	0.179	0.079	0.137	2.257*	0.02	1.81
	Empathy toward natural environments	0.211	0.082	0.154	2.575*	0.01	1.75
	Awareness of natural environments	−0.027	0.050	−0.027	−0.550	0.58	1.15
	*F*-value= 10.737***	*R* = 0.256	R^2^ = 0.066	Adj. R = 0.060			

***p < 0.001, **p < 0.01, *p < 0.05.

#### 3.3.2 The association between nature connection and children’s well-being

Children’s well-being is increasingly a focus of societal concern. Studies indicate that nature has therapeutic benefits that contribute to personal fulfillment. Table 5 shows that the three aspects of nature connection - ‘contact with natural environments, “empathy toward natural environments, ‘and ‘awareness of natural environments’ - significantly impact children’s well-being (*F* value = 60.053, *p* < 0.001), explaining 27.6% of the variance. Notably, both ‘contact with’ and ‘empathy toward’ natural environments are positively correlated with increased well-being (β = 0.357 and *β* = 0.236, respectively, both *p* < 0.001), while ‘awareness of natural environments’ did not show a significant effect (*p* > 0.05).

**Table 5:** Nature connection on children’s well-being.

Independent variable	Unstandardized regression coefficient (B)	S.E	Regression coefficient (β)	*t*	*p*-value	VIF
Constant	1.832	0.157		11.680***	<0.001	
Nature connection						
Contact with natural environments	0.312	0.046	0.357	6.730***	<0.001	1.81
Empathy toward natural environments	0.217	0.048	0.236	4.524***	<0.001	1.75
Awareness of natural environments	−0.025	0.029	−0.036	−0.852	0.39	1.15
*F*-value = 60.053***	*R* = 0.530	R^2^ = 0.281	Adj. R = 0.276			

***p < 0.001, **p < 0.01, *p < 0.05.

#### 3.3.3 Exploring the role of natural environments in children’s social and emotional development: themes and subthemes from qualitative interviews

Table 6 illustrates two main themes: “Enjoyment of Natural Spaces” and “Therapeutic Benefits, “which reveal the positive impact of natural environments on children’s social and emotional well-being. The “Enjoyment of Natural Spaces” theme highlights how children derive joy and engagement through peer interaction and individual activities, such as playing in parks or collecting natural objects like cicada shells. The second theme, “Therapeutic Benefits, “emphasizes how natural spaces provide emotional respite, interaction with animals, and a sense of peace, contributing to children’s emotional calm and happiness. This table underscores how these elements foster both social bonds and emotional health in children.

**Table 6:** Themes and subthemes illustrating the impact of natural environments on children’s social and emotional well-being.

Main theme	Subtheme	Description
Enjoyment of natural spaces	Peer interaction	Children find joy in playing with peers in natural settings, such as parks.
	Individual activities	Activities like collecting cicada shells provide enjoyment and engagement with nature.
Therapeutic benefits	Emotional respite	Natural spaces offer a retreat where children can feel at ease and temporarily escape from emotional distress.
	Interaction with animals	Animals in natural settings, like a dog in the park, provide companionship that enhances emotional well-being.
	Sense of peace	Quiet and tranquil natural settings, like forests, contribute to emotional calm and happiness.

## 4 Discussion

The results of this study provide insights into the roles of pet ownership and nature connection in children’s lives. Hypothesis H1, which explored the relationship between pet ownership and variations in children’s connection to nature, was partially supported. Our variance analysis identified significant differences specifically in the domain of ‘empathy toward natural environments (25). However, no significant differences were observed in ‘contact with natural environments’ or ‘awareness of natural environments’ (25). This suggests that while pet ownership may enhance children’s empathetic responses to nature, it does not appear to affect their direct interaction or cognitive awareness of natural environments (26). These findings align with previous research indicating that empathy for nature can be influenced by various factors, including personal experiences and educational interventions (27). For example, studies have shown that children with pets often demonstrate higher levels of empathy and nature emotional connection to animals and nature (21, 28, 29). Additionally, educational programs focusing on nature and empathy have been found to enhance children’s emotional responses to natural environments (6). Further investigation is needed to fully understand the underlying mechanisms and potential implications of these relationships. In contrast, Hypothesis H2, which proposed that pet ownership status would be significantly associated with children’s interpersonal relationships, was not confirmed. Our analysis did not reveal significant differences in the interpersonal relationships of children based on their pet ownership experiences. This result contrasts with the findings of Koyasu (30), who observed significant associations in older adolescents and university students. It is possible that the length of time children has owned pets could also impact their interpersonal relationships, a variable we did not measure in this study (31). Future research should consider including the duration of pet ownership as a factor to better understand its potential influence on social interactions. Additionally, developmental stages play a crucial role, as younger children may have different social dynamics compared to older individuals with more mature caregiving experiences (24, 32–33). For instance, longer pet ownership may provide more opportunities for children to develop and refine their social skills through sustained interactions with pets (34–36). Including this variable in future analyses could provide a more nuanced understanding of how pet ownership affects interpersonal relationships across different age groups.

Hypothesis H3, which proposed that children’s pet ownership status would significantly influence their sense of well-being, was not supported in our study. The variance analysis revealed no significant differences in well-being based on children’s pet ownership experiences. This contrasts with Matijczak et al.'s (37) findings, which suggested that positive interactions with pets stabilize emotions and provide support similar to human social connections. The discrepancy may be attributed to the age and independence of the participants; our study focused on children as opposed to Matijczak’s university-aged subjects. During interviews about well-being, children’s responses varied significantly. Some expressed happiness due to having more than children in impoverished situations, while others believed that family companionship would enhance their happiness, suggesting that family presence might be more significant than pet-ownership for children (38–41).

*H4*: There is a positive association between children’s connection to nature and their interpersonal relationships. Regression analysis examining the influence of children’s nature connection on their social interactions revealed that both ‘contact with’ and ‘empathy toward’ natural environments significantly affected all aspects of interpersonal relationships.

This finding is similar to Colding et al.'s (42) results, suggesting a positive correlation between nature connection and social relations.

Hypothesis H5, proposing that a connection to nature is associated with enhanced well-being, was supported. Our regression analysis examined the correlation between children’s connection to nature and their sense of well-being. The findings indicated that both ‘contact with natural environments’ and ‘empathy toward natural environments’ significantly contribute to children’s happiness. This aligns with the research by Mohamada (43), which found that children use their favorite natural settings for emotional regulation, thereby enhancing their psychological health. In interviews, children echoed this sentiment, affirming nature’s healing properties.

The study showed that children with pet ownership experience had significantly higher scores in ‘empathy toward natural environments’ on the nature connection scale compared to those who had never owned pets. Children who had cared for pets exhibited more familiarity and less fear or apprehension toward animals, leading to greater empathy for animals in natural environments. Both ‘contact with natural environments’ and ‘empathy toward natural environments’ in nature connection demonstrated strong predictive power for various aspects of interpersonal relationships. Past research, such as Barrera-Hernández et al. (44) and Barrable and Booth (45), supports our findings, highlighting the positive social impact of children’s engagement with natural environments. Quantitative analysis and qualitative interviews revealed that children’s interaction with nature facilitated relaxation and satisfaction, thereby enhancing their sense of well-being. This aligns with international research supporting the idea that natural environment engagement contributes to children’s happiness (46–48).

## 5 Conclusion

This study boasts three significant strengths. First, a mixed-methods approach was employed, combining qualitative and quantitative research methodologies. This approach facilitated a comprehensive exploration of the impact of children’s pet-ownership on both natural connection and interpersonal relationships from various angles. Qualitative re-search captured emotions, motivations, and underlying stories, while quantitative re-search provided data support. The mutual validation between the two methods enhanced the credibility and comprehensiveness of the study’s findings. Second, multidimensional analysis: This study conducted multi-level analyses of children’s pet-ownership, natural connection, and interpersonal relationships. It delved deeply into the relationships be-tween these factors from different dimensions. This multidimensional analysis enhanced the understanding of the complexities inherent in these issues, offering more specific in-sights and conclusions. Third, high questionnaire effectiveness: During on-site surveys in Taoyuan City, 471 valid questionnaires were successfully collected, yielding an effective utilization rate of 96.71%. This demonstrates the proactive cooperation of participating students and schools, enhancing the representativeness and reliability of the research findings. This consequently lends greater persuasiveness to the study’s results.

## 6 Strengths and limitations

Our study suggests that interactions with pets can significantly enhance children’s emotional well-being and interpersonal relationships. Health practitioners, especially those working with children, can incorporate animal-assisted therapies or recommend pet interactions as part of holistic health interventions (49). These practices could be particularly beneficial in developmental and therapeutic settings to improve social skills, reduce stress, and enhance emotional expression among children.

The positive association between pet ownership, natural connection, and child well-being underscores the need for supportive policies that facilitate pet ownership and protect natural environments. Policy practitioners could use this evidence to advocate for urban planning that includes pet-friendly spaces and initiatives that encourage interaction with nature. Furthermore, educational policies could be adapted to include more nature-based and animal-assisted learning activities, recognizing their benefits in promoting healthier, happier, and more connected communities.

This study opens several avenues for future research. Researchers can explore the long-term impacts of pet interactions across different developmental stages, investigate the effects of different types of pets, or examine the psychological and physiological mechanisms underpinning the benefits of pet ownership. Additionally, longitudinal studies could assess how sustained interactions with pets and nature influence health outcomes over time.

This research has certain limitations. While the study’s focus on fifth and sixth-grade students in public elementary schools within Taoyuan City was intentional and aligns with our research design, future research could benefit from expanding the scope to include different regions and broader age groups, including adolescents and college students from junior and senior high schools. This would help in understanding the effects of pet interaction across a more diverse developmental spectrum. Additionally, adopting a longitudinal approach could provide deeper insights, allowing for the collection of data through long-term tracking to comprehensively verify the impact of pet ownership and interaction behavior on natural connection, interpersonal relationships, and happiness. Additionally, this study’s focus on pet ownership as a variable did not account for the depth and nature of the interactions between children and their pets. The variable was limited as it provided no insights into the quality or frequency of these interactions, which can vary greatly among individuals. Future studies should consider more detailed measures of interaction to better understand how varying levels of engagement with pets affect children’s development and well-being.

The modifications made to two measurement tools in this study may also impact the validity of these measures. Details regarding how these changes could affect the research findings should be considered and discussed further in subsequent studies.

Furthermore, the absence of control variables in the quantitative analysis is a notable limitation. Important confounding factors such as race/ethnicity, gender, pet species, mental health symptoms, parent education, and socioeconomic status were not controlled for, which might have influenced the outcomes. Future research should include these variables to provide a clearer picture of the relationships being studied.

Lastly, while the qualitative interviews provided additional context to the quantitative data, the depth of exploration was limited due to time constraints. Future researchers are encouraged to employ more immersive techniques such as worksheets, drawings, or individual in-depth interviews to delve deeper into the intricate relationships and influences among children’s pet ownership, their natural connection, interpersonal relationships, and happiness. These measures would contribute to a more comprehensive development of the research in this field.

## Data Availability

The original contributions presented in the study are included in the article/Supplementary material, further inquiries can be directed to the corresponding author.
