# Supplementary material for: Peer bonds and nature’s embrace: exploring the influence of pet caregiving on social well-being and nature connection among Taiwanese children
Source: Front Public Health. 2024 Oct 21;12:1431939. doi: 10.3389/fpubh.2024.1431939 (PMC11533891; doi:10.3389/fpubh.2024.1431939)
Supplement: Supplementary file 1 [file Table_1.DOCX]

Supplementary Material

**Table S1.** Content of the qualitative interview questionnaire

| Variable (question) | Measurement items |
| --- | --- |
| Pet Interaction Behavior | 1. What kind of pets did you have? What is your current pet? How long have you raised it?  2. Does your pet have any special meaning to you?  3. About how long do you spend with your pet in a day?  4. What do you do with your pet most often? what did you do for it Has it done anything for you? |
| natural connection | 1. What do you think is the natural environment? Can you give me an example?  2. Do you like to be in contact with the natural environment (parks, community gardens, etc. include natural landscapes)? How often do you usually go?  3. What do you usually do there?  4. What does it mean to you to be in contact with the natural environment? |
| interpersonal relationship | 1. Do you like school (home)? Why?  2. Do you usually prefer to be alone or play with your family (classmates)? Why?  3. What role do you think you play at home (school)? |
| Happiness | 1. Do you think you are happy now? Why?  2. How do you think changing your current situation can make you happier? (several factors may be listed) |
